# Neutralizing antibodies in the intestinal mucosa are essential to control gastrointestinal infection by Shiga toxin‐producing *Escherichia coli*

**DOI:** 10.1002/mlf2.70026

**Published:** 2025-08-25

**Authors:** Alan Mauro Bernal, Fernando Nicolás Sosa, Yina María Carpintero‐Polanco, Camila Dara Cancino, Romina Jimena Fernández‐Brando, María Victoria Ramos, Ariel Podhozer, Agustina Errea, Martín Rumbo, Marina Sandra Palermo

**Affiliations:** ^1^ Instituto de Medicina Experimental–CONICET–Academia Nacional de Medicina Ciudad Autónoma de Buenos Aires Argentina; ^2^ Instituto de Estudios Inmunológicos y Fisiopatológicos–CONICET–Universidad Nacional de La Plata La Plata Argentina

**Keywords:** antibodies, gastrointestinal infection, HUS, mouse model, STEC

## Abstract

Infections with Shiga toxin (Stx)‐producing *Escherichia coli* (STEC) strains can result in a wide range of clinical presentations. Despite STEC O157:H7 being the serotype most frequently associated with hemolytic uremic syndrome (HUS), in some patients, a self‐limited gastrointestinal infection is observed. We have previously demonstrated that genetic differences between BALB/c and C57BL/6 mice account for a different outcome after an experimental gastrointestinal STEC O157:H7 infection, in which the better outcome observed in BALB/c mice was associated with a Th‐2 biased immune response. The objective of this study was to determine the role of anti‐STEC antibodies during STEC O157:H7 infections. We first demonstrated that the B‐cell‐dependent response triggered upon STEC O157:H7 infection is necessary to keep BALB/c mice healthy and reciprocally C57BL/6 mice pre‐challenged with an Stx2‐deficient STEC O157:H7 strain were able to survive, remaining healthy after a subsequent STEC O157:H7 infection. We further proved that anti‐STEC O157:H7 antibodies raised after infection have binding specificity against STEC O157:H7 bacteria, recognize H7, and have neutralizing capacitiy, by interfering with important pathogenic mechanisms such as motility and adhesion to intestinal epithelial cells. We conclude that local and/or systemic specific antibodies against STEC mediate prevention of lethal complications during STEC O157:H7 infections.

## Impact statement

Gastrointestinal infections caused by Shiga toxin‐producing *Escherichia coli* (STEC) can lead to self‐limited infections, but also to life‐threatening diseases, mainly hemolytic uremic syndrome (HUS). Although several variables may impact the outcome, we hypothesized that the capacity to develop an early and specific antibody response against virulence factors is the central defining component of host response. We demonstrated that systemic and/or local anti‐STEC antibodies, capable of recognizing specifically STEC O157:H7, can impair STEC motility and adhesion to intestinal epithelial cells. Thus, this study provides insight into the protective role that specific antibodies play in the prevention of systemic diseases secondary to STEC infections, providing new therapeutic options.

## INTRODUCTION

Hemolytic uremic syndrome (HUS) develops secondary to infections with Shiga toxin (Stx)‐producing *Escherichia coli* (STEC) strains and is characterized by microangiopathic hemolytic anemia, thrombocytopenia, and renal dysfunction. In Argentina, STEC O157:H7 is the serotype most frequently associated with HUS^1^. Although Stx is necessary for progression to HUS, its presence does not determine the development of syndrome because only a percentage of children who have gastrointestinal infections with STEC strains develop HUS^2^, ^3^. The humoral response in the intestine against pathogens such as STEC has been reported to be relevant in defining the outcome to self‐limited forms.

Antibodies (Abs) are considered central players during the management of infectious diseases^4^, ^5^. These Abs are recognized as anti‐infective neutralizing Abs. In addition, binding or opsonizing Abs are able to facilitate killing of the pathogen by effector cells, including Ab‐dependent cellular cytotoxicity, phagocytosis, and activation of the classical complement pathway, which depend on the Fc domain and subsequent interaction with Fc gamma receptors or with the components of the complement cascade^4^. Overall, anti‐infective Ab therapy offers prophylactic or therapeutic tools to fight against infections when vaccines and/or antibiotics are neither available nor efficacious^6^, ^7^, ^8^. In particular, Abs against toxins like Stx are able to block one of the major pathogenic factors among STEC infections^9^, ^10^, ^11^.

In addition, a comparative study on vaccine inoculation routes has shown that mucosal routes provide greater efficacy in inducing local immune responses compared to systemic administration of the same formulation as well as long‐term protection^12^. In particular, immunization with airway pathogens by the mucosal route induced high protection at local or even remote sites^8^, ^13^.

We have previously demonstrated that BALB/c mice present a much better outcome compared to C57BL/6 mice after STEC O157:H7 gastrointestinal infection^14^. Considering that BALB/c mice are prone to developing Th2‐biased immune responses, we hypothesized that this behavior is probably associated with an early and specific Ab response against pathogenic factors. Thus, the objective of this study was to determine if the B‐cell‐dependent response triggered upon STEC O157:H7 infection is necessary to keep BALB/c mice healthy. We confirmed that B lymphocyte‐depleted BALB/c mice died after STEC O157:H7 infection, showing Stx‐associated renal damage. Reciprocally, we demonstrated that susceptible C57BL/6 mice pre‐challenged with an Stx‐deficient STEC O157:H7 strain were protected from death and renal damage when they were subsequently infected with STEC O157:H7. We found specific anti‐STEC O157:H7 and anti‐flagellin H7 (H7) IgG and IgA in plasma and fecal supernatants from infected or pre‐challenged mice capable of binding to STEC O157:H7, but not commensal *E. coli*. Subsequently, we demonstrated that these Abs are able to impair STEC O157:H7 motility and adhesion to intestinal epithelial cells. We conclude that local and systemic specific anti‐STEC O157:H7 Abs mediate protection against systemic complications and death during STEC O157:H7 infections.

## RESULTS

### Survival rates and disease severity in BALB/c mice with or without B lymphocyte depletion

To study the role of a specific B‐dependent response in STEC O157:H7‐infected BALB/c weaned mice, we administered anti‐B220 Ab (2.8 mg/mouse). Then, the percentage of CD19 positive cells (%CD19^+^) in mesenteric lymph nodes (MLNs) and the spleen was analyzed by flow cytometry at different time points (4, 24, and 48 h) to confirm B‐cell depletion. As shown in Figure S1, this treatment induced significant B‐cell depletion in spleens and MLNs compared to controls at all time points evaluated (Figure S1); however, at 48 h, the %CD19^+^ cells in spleens started to increase.

**Figure 1:**
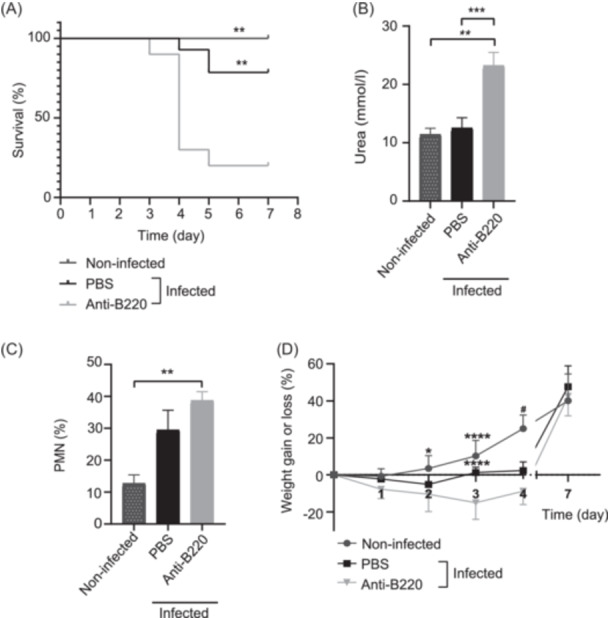
Survival rates and parameters associated with systemic disease after infection of BALB/c mice with or without B‐lymphocyte depletion. BALB/c mice treated with anti‐B220 Ab (*n* = 10) or PBS (*n* = 14) were simultaneously infected with >1.5 × 10^10^ CFU of STEC O157:H7/mouse as described in the Materials and Methods. Non‐infected littermates were used as controls (*n* = 5). The animals were observed and weighed daily until Day 7, when survivors were euthanized. On 3 dpi, mice were bled to obtain whole blood and plasma. Each value represents the mean ± SEM. (A) Survival rates. Data were analyzed using the Mantel–Cox test; **p < 0.01 compared to the anti‐B220‐treated group. (B) Plasma urea levels. (C) Percentage of polymorphonuclear leukocytes (PMNs) in whole peripheral blood. Data were analyzed by one‐way ANOVA using Tukey's posttest. **p < 0.01; ***p < 0.001. (D) Percentage of weight gain or loss based on the initial weight. Data were analyzed by two‐way ANOVA using Tukey's posttest. *p < 0.05 compared to anti‐B220, ****p < 0.0001 compared to anti‐B220, and ^#^ *p* < 0.0001 compared to PBS and anti‐B220. dpi, days post infection.

After that, anti‐B220‐treated or PBS‐treated mice were intragastrically infected with infective doses higher than 1.5 × 10^10^ CFU of STEC O157:H7/mouse, and body weight and mortality were monitored daily. B‐depleted mice showed increased mortality rates starting 3 days post infection (dpi), compared to PBS‐treated mice and non‐infected mice (Figure 1A). B‐depleted mice showed biological signs of systemic disease (like HUS), such as high urea levels and neutrophilia (Figure 1B,C), while PBS‐treated mice did not differ significantly from non‐infected controls. Also, B‐depleted mice presented significant weight loss starting 2 dpi, in comparison with non‐infected mice (Figure 1D).

We conclude that B‐cell stimulation and the consequent Ab response play a central role in protection against STEC O157:H7 infections.

### Systemic and local humoral immune response in infected BALB/c mice treated or not with anti‐B220

**Figure 2:**
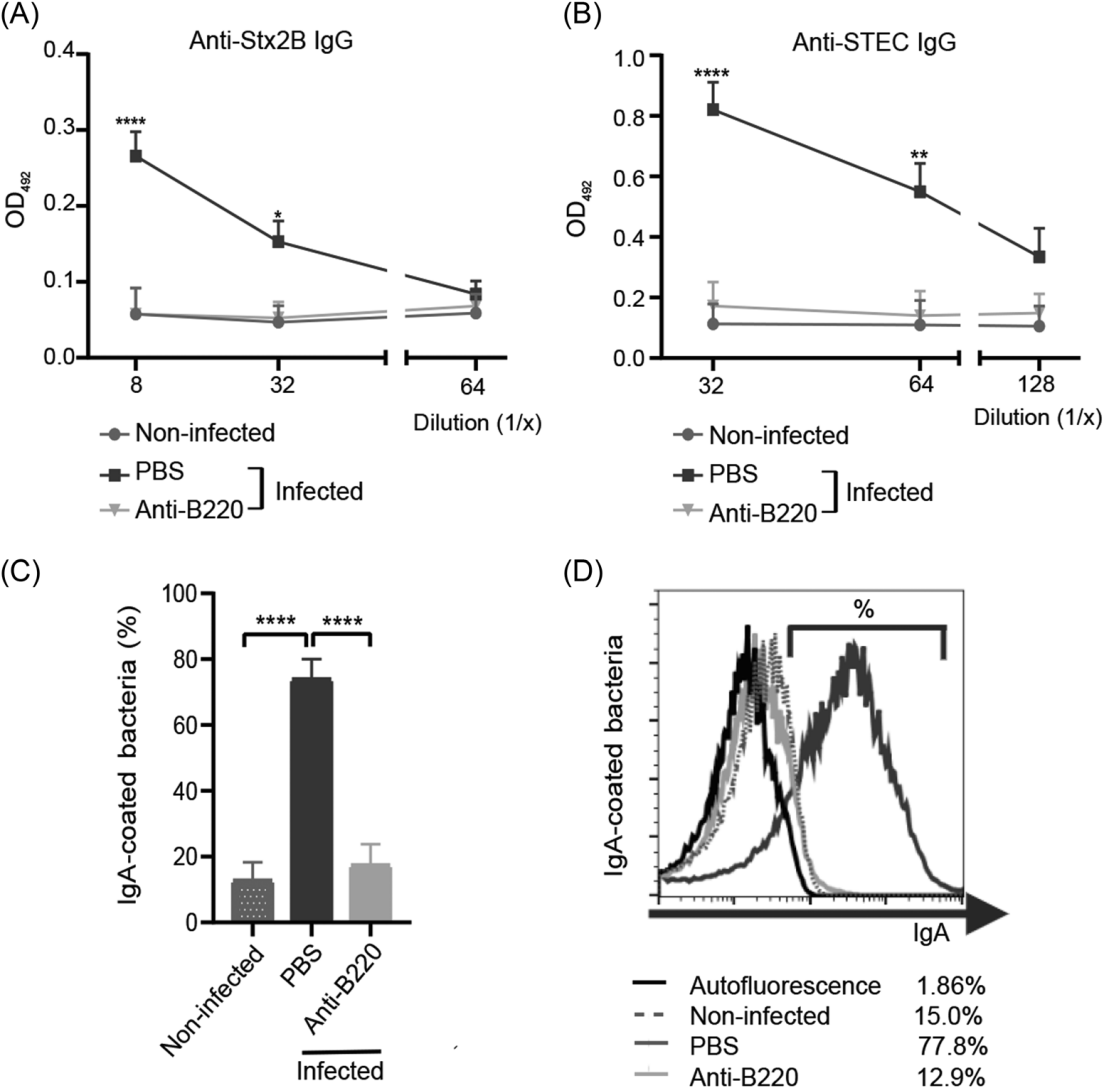
Systemic and local humoral immune responses in infected BALB/c mice treated or not with anti‐B220. The values in (A–C) shows the mean ± SEM of 4–8 mice/treatment. Titers of anti‐Stx2B (A) and anti‐STEC O157:H7 IgG (B) were determined in plasma at 3 dpi by ELISA. Anti‐Stx2B (A) and anti‐STEC O157:H7 (B) IgG titers were determined in plasma at 3 dpi by ELISA, based on the last dilution of the sample that yielded an OD_492_ significantly higher than those of the controls. Data were analyzed by two‐way ANOVA using Tukey's posttest. *p < 0.05, **p < 0.01, ****p < 0.0001 compared to infected BALB/c treated with anti‐B220 and non‐infected mice at the same plasma dilution. (C, D) Percentage of IgA‐coated bacteria in feces at 3 dpi assayed by flow cytometry. Feces from all groups were incubated or not with FITC‐coupled anti‐mouse IgA (C), as described in the Materials and Methods. Data were analyzed using a one‐way ANOVA, followed by Tukey's posttest. ****p < 0.0001. Representative histograms (D) show unstained samples (autofluorescence, black line) and FITC anti‐mouse IgA‐stained samples from non‐infected (gray dotted line) and infected mice treated with PBS (dark gray line) or with anti‐B220 Ab (light gray line) assayed in parallel.

To confirm that B lymphocyte depletion abrogated specific Ab generation, we tested Ab levels against relevant bacterial virulence factors, such as Stx2 and antigens expressed on the bacterial surface. As expected, B‐depleted mice did not show significant levels of anti‐Stx2 or anti‐STEC O157:H7 IgG in plasma (Figure 2A,B), in sharp contrast with PBS‐treated infected mice. To assay the production of specific anti‐STEC O157:H7 IgA in mucosa, we determined the percentage of IgA‐coated bacteria in feces of anti‐B220‐treated or PBS‐treated mice at 3 dpi by flow cytometry. As can be observed in Figure 2C,D, the PBS‐treated infected group was the only group that showed a significant high percentage of IgA‐coated bacteria. These results demonstrated that anti‐B220 treatment was effective in depleting the B lymphocyte population in mice, and therefore, those mice were not capable of generating a specific humoral immune response.

### Survival rates and disease severity in C57BL/6 mice pre‐challenged or not with a nonlethal Stx2‐deficient STEC O157:H7 (ΔStx2) strain

**Figure 3:**
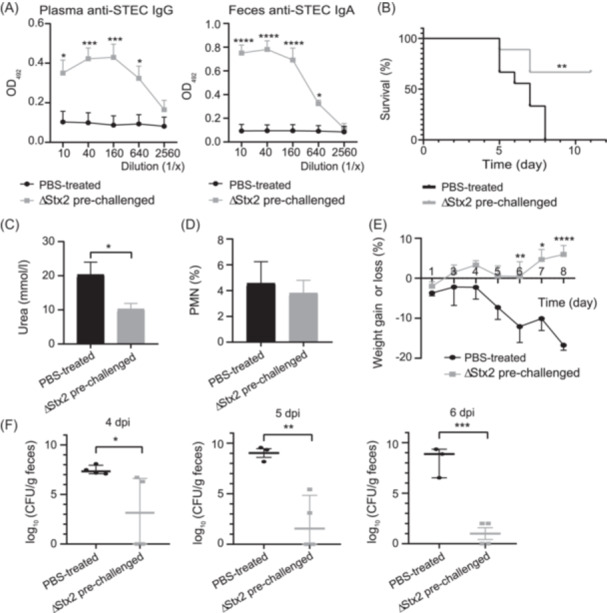
Protection capacity of anti‐STEC O157:H7 Abs. C57BL/6 mice were intragastrically inoculated twice with ΔStx2 or PBS at 10‐day intervals, as described in the Materials and Methods. (A) Determination of anti‐STEC IgG and IgA. Seven days after the second challenge, plasma and feces were collected to determine anti‐STEC O157:H7 IgG (left) and IgA (right) titers, respectively, based on the last dilution of the sample that yielded an OD_492_ significantly higher than those of the controls. Data were analyzed by two‐way ANOVA, followed by Tukey's posttest (*n* = 9). *p < 0.05, ***p < 0.001. Ten days after the second challenge, mice were infected with the pW strain, as described in the Material and Methods. Mice were observed daily to confirm mortality, weighed, and bled at 6 dpi, at which time point the % PMN in whole blood and plasma urea levels were measured. Also, from 4 to 6 dpi, feces were collected to determine the number of pW CFUs per gram of feces (expressed on a logarithmic scale). (B) Survival rates. Data were analyzed using the Mantel–Cox test (*n* = 9). **p < 0.01. (C) Plasma urea levels. (D) % PMN in whole blood. Data were analyzed by one‐way ANOVA, followed by Tukey's posttest (*n* = 6). *p < 0.05. (E) Percentage of weight gain or loss based on the initial weight. Data were analyzed by two‐way ANOVA, followed by Tukey's posttest (*n* = 9). *p < 0.05, **p < 0.01, ****p < 0.0001. (F) Bacterial shedding. Data were analyzed using a non‐parametric (Mann–Whitney; *n* = 4) or parametric *t* test (Student's *t* test; *n* = 3 − 4), as appropriate. *p < 0.05; **p < 0.01; ***p < 0.001. Each value represents the mean ± SEM in all graphs, except panel (F) (4 dpi), which shows the median ± interquartile range.

The above results demonstrated that BALB/c weaned mice are protected from a lethal STEC O157:H7 infection through a B‐cell‐dependent immune response, supporting previous data^14^. Considering that anti‐Stx2 Abs have been widely demonstrated to be efficient in neutralizing Stx2 and thus preventing STEC‐associated mortality^11^, ^14^, we wanted to determine the role that anti‐STEC O157:H7 Abs may play in providing protection. To achieve this, susceptible C57BL/6 mice were challenged with two doses of a nonlethal ΔStx2 strain. Seven days after the second challenge, mice were bled and their feces were collected to confirm the presence of specific anti‐STEC O157:H7 Abs. These mice presented significant high levels of anti‐STEC O157:H7 IgG and IgA in plasma and feces, respectively (Figure 3A). Subsequently, ΔStx2 pre‐challenged or PBS‐treated control mice were treated with ampicillin and then infected with an Stx2‐producing *E. coli* serotype O157:H7 strain pW, which carries a plasmid with ampicillin resistance, because adult mice should be depleted of commensal bacteria to make them susceptible to STEC pathogenicity^15^, ^16^.

As shown in Figure 3, PBS‐treated mice showed a significant increase in mortality, plasma urea levels, and loss of body weight, and a slight increase in the percentage of neutrophils compared to ΔStx2 pre‐challenged mice (Figure 3B–E). In addition, when we evaluated the pW shedding, we found significantly higher numbers of pW CFU in feces from PBS‐treated mice compared to ΔStx2 pre‐challenged infected mice at all time points evaluated (Figure 3F).

We conclude that specific anti‐STEC O157:H7 Abs are enough to confer protection to sensitive mice from systemic complications following STEC O157:H7 infection and, afterward, we investigated the mechanisms by which STEC O157:H7‐specific Abs could impair STEC pathogenicity.

### Characterization of anti‐STEC O157:H7 IgG and IgA from plasma and feces of infected mice

In order to evaluate the functional capacity of anti‐STEC O157:H7 Abs, BALB/c mice were infected multiple times to achieve high titer of anti‐STEC O157:H7 Abs in plasma and feces. We chose this mouse strain because it presents a Th2‐biased immune response, driving high titers of specific Abs. The infection scheme is clearly illustrated in Figure S2A.

As shown in Figure S2B, circulating anti‐STEC O157:H7 IgG significantly increased in plasma of BALB/c mice after each challenge with STEC O157:H7. Moreover, the anti‐STEC O157:H7 IgA titer reached a value of 1024 in feces (defined as the last sample dilution that presents an OD_492_ that is higher and statistically significantly different from that of the control samples, Figure S2C) at the end of the experiment. These results confirm significant and specific systemic and mucosal Ab production after STEC O157:H7 infection in BALB/c mice and show that specific IgG increased along with reinfections.

**Figure 4:**
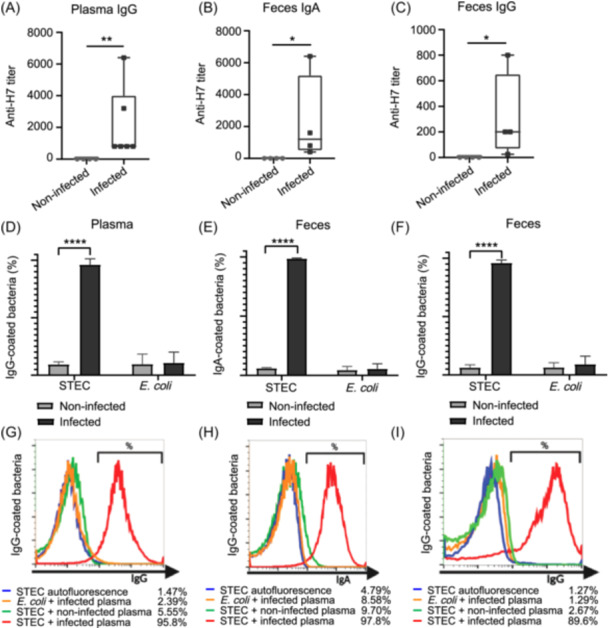
Characterization of IgG and/or IgA of plasma and feces from infected BALB/c mice. (A–C) Anti‐H7 IgG/IgA titer in plasma (A) and feces supernatants (B, C). BALB/c mice were bled, and plasma and feces were collected on Day 7, after the last infection dose, to determine anti‐H7 titer. The values represents the median ± the interquartile range. Data were analyzed using a non‐parametric *t* test (Mann–Whitney; *n* = 4 − 6). *p < 0.05; **p < 0.01. (D–F) Opsonizing capacity of anti‐STEC O157:H7 Abs present in plasma and fecal supernatants against STEC and *E. coli* bacteria. 10^7^ CFU of STEC O157:H7 or 10^7^ CFU of commensal *Escherichia coli* were incubated with a 1/10 dilution of plasma (D) or with a 1/2 dilution of fecal supernatants (E, F) from infected or non‐infected BALB/c mice for 18 h. After incubation, the percentage of IgA‐ or IgG‐coated bacteria was determined by flow cytometry using a FITC‐coupled anti‐mouse IgA or IgG. The values represents the mean ± SEM of three biological replicates. Data were analyzed by two‐way ANOVA, followed by Tukey's posttest. ****p < 0.0001. (G–I) Representative histograms of STEC incubated with plasma (G) or fecal supernatants (H, I) from infected (red line) or non‐infected (green line) BALB/c mice. Autofluorescence was measured by acquiring the corresponding bacteria incubated without any primary or secondary Abs (blue line). As commensal *E. coli* autofluorescence resembles STEC O157:H7 autofluorescence, only STEC O157:H7 autofluorescence was depicted. For each histogram, *E. coli* incubated with plasma (G) or fecal supernatants (H, I) from infected BALB/c mice were depicted as an orange line.

Considering that H7 is the protein structural subunit of the flagellum, which is not only the motility apparatus of bacteria but also plays a role in adherence of bacteria to host cells^17^, we assayed the presence of anti‐H7 Abs in plasma and feces from infected mice. High titers of anti‐H7 IgG and IgA were detected in plasma and feces from infected mice (Figure 4A–C).

To further characterize the specific IgG and IgA present in the plasma and feces of infected mice, we determined whether they were able to specifically recognize STEC O157:H7 but not commensal *E. coli*. As shown in Figure 4D–I, anti‐STEC O157:H7 IgG and IgA detected in plasma and feces recognized and opsonized STEC O157:H7 specifically, without binding to commensal *E. coli*. In contrast, plasma or feces from non‐infected BALB/c mice did not present Abs (IgG or IgA) capable of recognizing STEC or commensal *E. coli*.

### Evaluation of the neutralizing capacity of anti‐STEC O157:H7 IgG from the plasma of infected mice

Having observed the presence of IgG with opsonizing capacity in feces and plasma, and considering that part of the fecal IgG is the result of receptor‐mediated transcytosis of plasmatic IgG across the epithelial barrier^18^, ^19^, we decided to carry out in vitro neutralization assays using only plasma obtained from infected or non‐infected mice, since it is a sterile sample.

**Figure 5:**
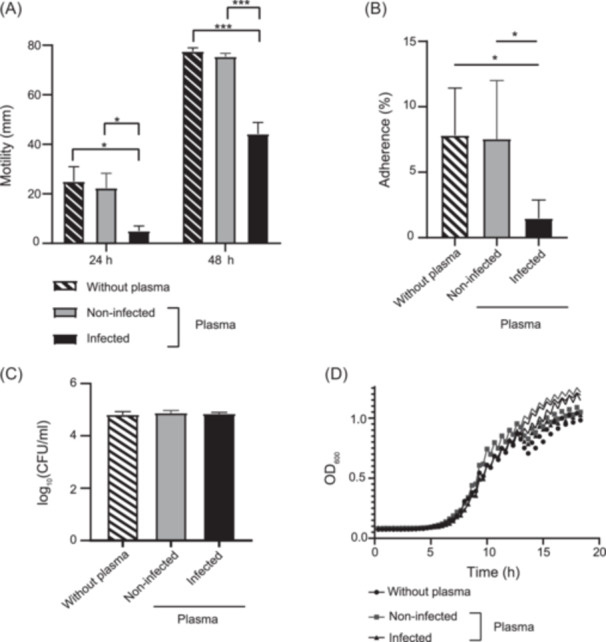
Neutralizing capacity of plasma from infected BALB/c mice. STEC O157:H7 bacteria were incubated with plasma (1/10 dilution) from infected or non‐infected mice, or left to incubate without plasma, for 18 h and different bacterial properties were assayed as described in the Materials and Methods. (A) Bacterial motility at 24 and 48 h. The values represent the mean ± SEM of five biological replicates. Data were analyzed by two‐way ANOVA, followed by Tukey's posttest. *p < 0.05; ***p < 0.001. (B) Adhesion to Caco‐2 epithelial cells after 3 h of infection. Each value represents the mean ± SEM of four biological replicates. Data were analyzed using the Kruskal–Wallis test. *p < 0.05. (C) Initial bacterial count (time 0). Data were analyzed by one‐way ANOVA, followed by Tukey's posttest. Each value represents the mean ± SEM of four biological replicates. (D) Bacterial growth recorded for 18 h using a multimode plate reader as OD_600_. Data were analyzed by two‐way ANOVA, followed by Tukey's posttest for multiple comparisons. Each value represents the mean ± SEM of four biological replicates.

Thus, we evaluated the neutralizing capacity of anti‐STEC O157:H7 Abs in terms of bacterial motility and adhesion to intestinal epithelial cells, two important properties of pathogenic bacteria to induce intestinal damage^20^. First, we studied whether the Abs produced in infected mice could inhibit bacterial motility in vitro at 24 and 48 h after seeding in soft agar plates. As shown in Figures 5A and S3, bacterial motility was significantly reduced upon incubation with plasma from infected mice compared to plasma from non‐infected mice at both time points. Then, STEC O157:H7 bacteria were incubated with plasma obtained from infected or non‐infected mice and the number of adherent bacteria to Caco‐2 intestinal epithelial cells was calculated. Plasma from infected mice significantly reduced the percentage of STEC O157:H7 that adhered to intestinal epithelial cells compared to plasma from non‐infected mice (Figure 5B).

To evaluate inhibition of bacterial growth, the same number of viable STEC O157:H7 was first incubated with plasma from infected or non‐infected mice and then grown at 37°C under agitation, while recording OD_600_ every 20 min for a period of 18 h. No significant differences were observed in the viable count at time zero, thus assuming that both treatments started from the same number of STEC O157:H7 CFU. Furthermore, we did not observe differences in OD_600_ between both treatments at any of the time points evaluated within the 18 h period (Figure 5C,D), thus confirming that bacterial growth is not affected by anti‐STEC O157:H7 Abs.

We conclude that specific anti‐STEC O157:H7 Abs (present in plasma and feces from infected mice) are capable of limiting STEC O157:H7 pathogenicity through their binding and neutralizing capacity, which leads to impairment in bacterial motility and adhesion.

## DISCUSSION

Despite considerable advances in the understanding of pathogenic mechanisms of STEC strains and HUS development, treatment of *E. coli* O157:H7 infections, the STEC strain that is more commonly associated with HUS disease, remains challenging. Recently, development of treatments against infectious diseases based on specific antibodies has been receiving increasing attention^5^, ^8^. We hypothesized that knowledge of the precise nature of immune response associated with a better outcome and resolution of STEC infections could enable development of potential therapeutic interventions.

Overall, the results presented in this study indicate that the induction of specific Abs during STEC O157:H7 infection is a central component of immune response that protects mice against systemic complications and lethal effects secondary to STEC infections. Although several treatments based on anti‐Stx Abs have been developed and some of them are currently being assayed in clinical trials^9^, ^21^, the major drawback of these approaches is that they are applied when HUS is diagnosed and this is probably too late in relation to progression of the pathogenic cascade.

Herein, we showed that B lymphocyte depletion in BALB/c mice completely abolished anti‐STEC O157:H7‐specific Ab production and these mice die due to STEC O157:H7 infection with high levels of urea, neutrophilia, and significant weight loss, all of which are hallmarks of renal damage and Stx2‐associated HUS. These results suggest that the STEC tolerance observed in BALB/c mice is related to the triggering of a specific and rapidly initiated humoral immune response. Additional studies will be required to define which effector T‐cells are necessary for the induction of anti‐STEC immunity. In this regard, it has been reported that both B lymphocytes and CD4^+^ T lymphocytes play a central role in protection against gastrointestinal infections caused by *Citrobacter rodentium*, which is a murine pathogen that shares similar pathogenic characteristics to STEC O157:H7, given that both are LEE^+^ microorganisms that cause A/E lesions^22^.

This humoral immune response may include Abs against different bacterial pathogenic factors, mainly Stx2 and antigens expressed on the bacterial surface, such as bacterial lipopolysaccharide (LPS), type 3 secretion system (T3SS) components, and flagellin. Since the protective efficacy of antiserum against Stx1/Stx2 has been demonstrated in different preclinical models^11^, ^14^, ^23^, and in order to specifically evaluate the role of antibacterial Abs, we challenged susceptible C57BL/6 mice with an ΔStx2 to assess the protective capacity of anti‐STEC antibodies, excluding interference by anti‐Stx2 Abs, which are known to be highly protective. Through this approach, we first showed that antibacterial IgG and IgA were generated to a significant extent after two challenges with ΔStx2. Moreover, these pre‐challenged mice survived subsequent infection with an Stx2‐producing STEC O157:H7 strain, and were able to control bacterial shedding and probably the intestinal bacterial load. The reduction in fecal shedding observed in pre‐challenged mice was consistent with previous studies in which immunization of mice or calves with T3SS components reduced STEC O157:H7 load in the cecum and its excretion in feces^24^, ^25^. Efficient mucosal IgA response^26^, and also the receptor‐mediated transcytosis of IgG across the epithelial barrier^18^, ^19^ may be responsible for conferring protection against STEC O157:H7 colonization. Considering that the oral–fecal route is the central transmission route contributing to the spread of STEC O157:H7, reduction in shedding will also have a relevant impact on STEC epidemiology. In addition, it is known that specific Abs are important immunomodulators because they could bridge innate and acquired, cellular, and humoral immune responses, which are functions exerted once the Ab binds to the antigen‐forming immunocomplexes^6^, ^27^. In turn, IgA has been demonstrated to allow the selective entry of IgA‐coated bacteria into Peyer's patches, promoting the activation of targeted local and systemic immune responses^28^. Virulence factors enable STEC to transiently colonize intestines, overgrow host defenses, and consequently damage host intestinal architecture and function. After this process, bacterial toxins, mainly Stx2, cross the intestinal barrier and reach systemic circulation. In contrast, when these virulence factors are blocked by specific Abs, this sequence of events is disrupted and systemic complications secondary to Stx2 do not occur.

To directly evaluate the effector functions of anti‐STEC O157:H7 Abs, we infected BALB/c mice three times with increasing amounts of STEC O157:H7, to achieve high titers of specific Abs in plasma that guarantee sensitization of a high number of STEC O157:H7 during in vitro incubation, before assaying different pathogenic mechanisms such as bacterial motility, epithelial adhesion, and growth. Interestingly, we observed a significant increase in anti‐STEC O157:H7 IgG levels in the plasma after secondary or tertiary infection, suggesting the induction of a memory specific humoral immune response.

We and others have previously reported that H7 flagellin is a strong mucosal antigen and, even in the absence of any additional adjuvant, was enough to trigger antigen‐specific T‐ and B‐cell responses in mice^29^, ^30^, ^31^. Although it was not possible to fully determine which bacterial components are the targets of the anti‐STEC O157:H7 Abs, we were able to demonstrate that these Abs specifically recognize at least the H7 protein.

Moving forward in terms of characterization of anti‐STEC Abs, we demonstrated the binding capacity of plasma IgG, as well as fecal IgG and IgA, from infected mice against STEC O157:H7, but not against commensal *E. coli*. These results confirm the specificity of the immune response triggered and are in line with previous reports that demonstrated that a small number of highly transient exposures with competent bacteria robustly trigger highly effective immunity in mice, even in the absence of an inflammatory response^32^.

Afterward, we tested the Abs‐neutralizing capacity on three relevant bacterial mechanisms to induce pathogenicity: motility, adherence to intestinal epithelial cells, and growth. We used plasma samples instead of fecal supernatants from infected mice as a source of specific Abs, since they are sterile samples. For this reason, our assays essentially evaluated Abs belonging to the IgG class. Our results clearly showed the inhibiting activity of these Abs on motility and adherence, but we did not observe any inhibition of bacterial growth. We propose that inhibition of motility and adhesion is at least partially related to the presence of anti‐H7 Abs, since flagella have been identified as a major virulence factor of STEC O157:H7 involved in these bacterial functions^17^, ^33^, ^34^. In agreement with these findings, other authors reported that anti‐flagellin Abs may be responsible for inhibiting bacterial motility of *Salmonella* Typhimurium^35^ and *E. coli* strains, while anti‐*E. coli* LPS Abs partially reduce bacterial motility^36^. In turn, it has been reported that anti‐H7 Abs, in addition to interfering with motility, can downregulate the expression of genes involved in the synthesis of *E. coli* flagella^36^ and interfere with the adhesion of STEC O157:H7 to bovine rectal epithelial cells^33^. However, it is important to highlight that all these effects could be broader and stronger in vivo, since intraluminal‐specific IgA significantly contributes toward neutralizing several bacterial functions^37^. In fact, considerable evidence has demonstrated that intestinal IgA provides defense against bacterial enteropathogens by blocking virulence factors or by limiting bacterial interactions with intestinal epithelium, a general concept known as “exclusion function”^37^. Moreover, IgA‐mediated aggregation via classical agglutination or via growth inhibition of enchained daughter cells, which prevents their separation after division, leads to lower bacterial growth into the gut lumen and higher clearance^38^, ^39^. For this and other IgA functions, such as reduction of expression of bacterial pro‐inflammatory genes^40^ or reduction of host inflammatory response induced, that is, by H7, a known ligand for Toll‐like receptor five (TLR5)^41^, the neutralizing effects observed in vivo in the intestinal lumen could be broader and stronger than those evaluated in vitro by incubating bacteria with plasma (IgG), and are probably responsible for the complete protection observed in ΔStx2 pre‐challenged C57BL/6 mice infected with pW.

In turn, the presence of anti‐intimin, anti‐EspA, and anti‐EspB Abs was detected in patients with diarrhea or HUS due to STEC O157:H7 infections, evidencing the development of a specific humoral immune response during sickness and suggesting the relevance of these antibacterial Abs in protection^42^. Indeed, it is important to highlight that although there is a possibility of reinfections with strains of the same serotype or other STEC serotypes, typical HUS occurs only once^43^, suggesting that the Abs produced during a primary infection, as well as the immunological memory, are sufficient to prevent systemic complications during subsequent infections.

In conclusion, anti‐STEC O157:H7 Abs, through their binding and neutralizing capacity, interfere with bacterial motility and adhesion to intestinal epithelial cells and, as a consequence of this, are capable of limiting STEC colonization and pathogenicity, conferring protection to mice against systemic complications secondary to STEC infections.

## MATERIALS AND METHODS

### Bacterial strains

The enterohemorrhagic STEC strain used in this study was isolated from fecal specimens of a patient with HUS (STEC O157:H7)^44^. This strain belongs to the serotype O157:H7, and harbors the *eae*, *ehxA,* and *stx2a* genes, but not *stx1* ^45^. This strain was additionally transformed with a plasmid carrying only ampicillin resistance (pW)^46^.

For experiments assessing the protection associated with anti‐STEC O157:H7 antibodies, we used an isogenic strain of STEC O157:H7, in which the *stx2* gene had been deleted (ΔStx2)^47^.

To evaluate antibody specificity, we used commensal bacteria obtained as follows: feces were collected from a BALB/c mouse by spontaneous deposition at weaning. A 20 mg sample was cultured in 10 ml of tryptic soy broth (TSB) (Difco, Le Point de Claix, France) at 37°C for 24 h in a shaker. Then, 10 μl of this culture was streaked onto methylene blue eosin agar plates (Laboratorios Britania, CABA, Argentina) and incubated at 37°C for 24 h to isolate aerobic commensal bacteria. An *E. coli* colony was identified through biochemical tests and cultured in TSB to create new stocks of this commensal strain.

### Bacterial growth and preparation of stocks for mice infection

Single colonies of STEC O157:H7, ΔStx2, or pW were selected from lysogeny broth (LB) agar plates and cultured in 10 ml of TSB in a 37°C shaker until the exponential growth phase was reached. A 1/100 dilution of this culture was then transferred into an Erlenmeyer flask containing 50 ml of TSB for STEC O157:H7 and ΔStx2, or 10 ml of TSB for pW, and incubated overnight at 37°C. After centrifugation at 2000 rpm for 30 min, bacteria were washed twice and resuspended in phosphate‐buffered saline (PBS). The pW cultures were maintained with 100 μg/ml ampicillin.

A calibration curve was generated by plotting the number of STEC O157:H7, ΔStx2, or pW (CFU/ml) against OD_600_. For the mouse challenge, bacterial pellets of STEC O157:H7, ΔStx2, or pW were resuspended in PBS to obtain bacterial doses ranging from 1.0 × 10^11^ to 5.0 × 10^11^ CFU/ml for STEC O157:H7 and ΔStx2, or from 1.0 × 10^6^ to 5.0 × 10^6^ CFU/ml for pW, based on the calibration curve. The bacterial concentration was confirmed by plating serial dilutions on LB agar plates and counting the CFU.

### Mice

Mice (BALB/c and C57BL/6 strains) were originally purchased from Charles River Laboratory and bred under specific pathogen‐free (SPF) conditions in standard polypropylene cages and under controlled environmental conditions (temperature, 24 ± 2°C; humidity, 50% ± 10%; and 12 h light:12 h dark cycle) at the Animal Facility of the IMEX‐CONICET‐National Academy of Medicine, Buenos Aires, Argentina.

### Mouse models

#### B lymphocyte‐depletion scheme

BALB/c mice (sex indistinct) at weaning (17‐19 days old, 6‐10 g weight) were fasted for 4 h and then inoculated with an anti‐mouse B220 monoclonal Ab from ascitic fluid, kindly provided by Dr. Rumbo^48^. A single dose was inoculated intravenously to each mouse through the retro‐orbital plexus of 0.1 ml of 2.8 mg of anti‐B220 Ab. In parallel, PBS was administered to the control group of mice without B lymphocyte depletion. At 4, 24, and 48 h post inoculation, mice from both treatments were euthanized and the spleen and MLNs were removed for later processing and determination of the percentage of B lymphocytes by flow cytometry, as described below.

#### Infection of B lymphocyte‐depleted BALB/c mice at weaning with STEC O157:H7

BALB/c mice at weaning were randomly distributed into three groups: (1) group intravenously inoculated with PBS, but not infected (non‐infected), (2) group intravenously inoculated with PBS and infected (PBS), and (3) group intravenously inoculated with anti‐B220 Ab and infected (anti‐B220). The B lymphocyte‐depletion scheme consisted of intravenously inoculation through the retro‐orbital plexus of two daily doses of anti‐mouse B220 monoclonal Ab (0.1 ml containing 2.8 mg Ab/dose) spaced at 8 h for 3 days, to ensure total depletion of B lymphocytes. Each mouse from groups (2) and (3) was gastrointestinally infected after 4 h of fasting and 4 h after the first anti‐B220 inoculation, using a sterile stainless‐steel cannula (model 7.7.1; 0, 38 mm × 22 G, Harvard Apparatus, USA) containing 0.1 ml of a bacterial suspension of STEC O157:H7 (higher than 1.5 × 10^11^ CFU/ml PBS), achieving an infectious dose of >1.5 × 10^10^ CFU per mouse. Mice from non‐infected groups received PBS through the same route. Mice in all groups were observed daily to determine morbi‐mortality and on 3 dpi, blood samples were collected to perform differential blood cell counts using an automated hematologic counter (Abacus Junior Vet, Diatron, USA). Plasma samples were analyzed for urea concentration using a commercial kit (Urea Color Kit, Wiener Lab) and for anti‐STEC O157:H7 and anti‐Stx2B IgG levels by an enzyme‐linked immunosorbent assay (ELISA). Fecal samples were also collected at this time point for processing and determination of IgA‐coated bacteria.

#### Reinfection scheme of BALB/c mice

BALB/c mice at weaning and with 4 h of fasting were gastrointestinally infected by administering a nonlethal dose of STEC O157:H7 (1 × 10^10^ CFU/mouse) through a sterile stainless‐steel cannula. To increase the titer of anti‐STEC O157:H7 Abs, these mice were re‐challenged twice with STEC O157:H7 (5 × 10^10^ CFU/mouse) at 10‐day intervals. Mice were bled 7 days after the first and second infection to determine the titer of anti‐STEC O157:H7 Abs in the plasma. Seven days after the third infection, mice were euthanized to obtain plasma and feces and determine the anti‐STEC O157:H7 IgG and IgA titer, respectively. In parallel, a control group of BALB/c mice gastrointestinally inoculated with PBS at the same infection times was used for collection of plasma and feces without specific Abs (non‐infected group). Feces from large intestines were removed and diluted to 0.25 g/ml PBS containing 1 mM phenylmethylsulfonyl fluoride (PBS‐PMSF). Each sample was homogenized by vortexing for 5 min and centrifuged at 13,300 rpm for 5 min to precipitate bacteria. Fecal supernatants were collected, and free soluble IgA was analyzed by ELISA. The reinfected BALB/c mice are defined as infected BALB/c mice.

#### Immunization of C57BL/6 mice with ΔStx2 and later infection with pW

C57BL/6 mice at weaning and with 4 h of fasting were gastrointestinally infected with a bacterial suspension of ΔStx2 through a sterile stainless‐steel cannula (3.5 × 10^10^ CFU/mouse). Ten days after the first infection, mice received a booster by administration of 7.0 × 10^10^ ΔStx2 CFU/mouse. Seven days after the booster, mice were bled to obtain plasma, and their feces were collected by spontaneous deposition to determine the anti‐STEC O157:H7 IgG and IgA titer in plasma and feces, respectively, by ELISA (ΔStx2 pre‐challenged group). In parallel, a control group of C57BL/6 mice gastrointestinally inoculated with PBS at the same infection time points was used for collection of plasma and feces without specific Abs (PBS‐treated group).

Ten days after receiving the booster, all mice received ampicillin (2 mg/mouse) by oral gavage 6 h before infection and 18 h post infection as described by Bernal et al.^29^. For the infection, each mouse was inoculated with 0.1 ml of a bacterial suspension of pW containing 1.0–5.0 × 10^6^ CFU/ml, a dose range that results in 100% mortality by 8 dpi. Mice were monitored daily for weight and clinical signs until the conclusion of the experiments. On 6 dpi, blood samples were collected for differential blood cell counts and plasma urea determination. Feces were obtained by spontaneous deposition on 4, 5, and 6 dpi, and diluted to a concentration of 0.25 g/ml PBS. CFUs were determined by plating dilutions of the homogenized feces by vortexing onto MacConkey agar plates (Laboratorios Britania, CABA, Argentina) and incubating for 18 h at 37°C. Non‐sorbitol fermenting colonies were counted to determine pW shedding (expressed as CFU/g of feces).

### Obtention of leukocyte suspensions from the spleen and MLN

Spleens and MLNs of BALB/c mice were removed after 4, 24, and 48 h of treatment with anti‐B220 monoclonal Ab or PBS, and single‐cell suspensions were prepared by forcing the organs through sterile metal meshes. Single‐cell suspensions were washed once (0.5 g for 10 min at 4°C) with PBS and resuspended, in the case of splenocytes, in 2 ml of red blood cells lysis buffer (0.15 M NH_4_Cl, 10 mM NaHCO_3_ and EDTA 0.1 mM, pH = 7.4), and in the case of leukocytes from MLNs, in RPMI 1640 medium (Gibco, Invitrogen) with antibiotics (EMEVE Microvet SRL Laboratories). After 5 min of lysis, it was stopped by adding 10 ml of PBS, and the cell suspension was washed and finally resuspended in RPMI 1640 medium with antibiotics. The final suspensions of both organs were counted by Neubauer cameras to obtain the number of leukocytes per ml and perform flow cytometry assays.

### Immune response assessment by flow cytometry

#### B lymphocyte‐depletion measurement

Leukocytes obtained after processing the spleen and MLNs from BALB/c mice with or without B lymphocyte‐depletion (5 × 10^5^ cells in 0.2 ml RPMI 1640 medium with antibiotics), were incubated with an Ab against a cluster of differentiation that allows the identification of mouse B lymphocytes (CD19) coupled to FITC (0.8 µl/tube) (BD Biosciences) for 40 min at 4°C away from the light. The cell suspensions were washed with PBS and the pellet obtained was resuspended in 0.2 ml of 0.5% paraformaldehyde (PFA) for 30 min at 4°C, washed, and finally resuspended in 0.2 ml of PBS. Samples were acquired on a CyFlow Space cytometer (Sysmex Deutschland GmbH, Norderstedt, Germany). Leukocytes incubated without Abs (autofluorescence) were used as controls to set the cytometer settings. The lymphocytes were delimited based on their size (FSC) and granularity (SSC), and 20,000 events were acquired for subsequent analysis with FlowJo 10.0 software. The singlets were selected by exclusion using the FSC‐H versus FSC‐A and SSC‐H versus SSC‐A analysis. The percentage of CD19 positive events was calculated by establishing the autofluorescence of each sample as negative.

#### Determination of IgA‐coated bacteria

Feces from the large intestines were collected and diluted to 1 g/ml in PBS containing PMSF and were processed as previously described^14^. Briefly, each sample was sequentially centrifuged until the bacterial pellet was obtained and incubated with FITC‐conjugated rat anti‐mouse IgA (BD Biosciences) for 40 min at 4°C. Following incubation and washing, the bacterial pellets were resuspended in 2% PFA. Finally, the bacteria were washed again and finally analyzed for IgA‐coated bacteria using a FACSCalibur flow cytometer (BD Biosciences)^14^, ^49^. Bacteria were selected based on the size and granularity parameters (on a logarithmic scale for FSC and SSC), and 20,000 events were acquired per sample analyzed. An autofluorescence control was obtained for each sample, which was incubated without secondary Ab, to establish the cytometer settings. The results were analyzed using FlowJo 10.0 software, determining the percentage of bacteria coated with mouse IgA.

#### Determination of binding capacity of anti‐STEC O157:H7 Abs in vitro

STEC O157:H7 or commensal *E. coli* (1 × 10⁷ CFU/0.1 ml) were incubated with fecal supernatants (diluted 1/2) or plasma (diluted 1/10) obtained from non‐infected or infected BALB/c mice for 18 h at 4°C. The bacterial suspensions were then washed and incubated with FITC‐conjugated rat anti‐mouse IgG and/or IgA antibodies (BD Biosciences) for 40 min at 4°C away from the light. After incubation, the suspensions were washed, fixed in 2% PFA, and resuspended in 0.2 ml of PBS for acquisition using a FACSCalibur flow cytometer (BD Biosciences). Unstained STEC O157:H7 and commensal *E. coli* (without secondary antibodies) were analyzed to determine cytometer settings and autofluorescence levels. Bacteria were gated based on size and granularity (logarithmic FSC and SSC scales), and 20,000 events were recorded for analysis using FlowJo 10.0 software. The percentage of IgG or IgA bound to bacteria was calculated by using autofluorescence levels of each bacterium as the negative control.

### Assessment of immune response by ELISA

#### Determination of anti‐STEC O157:H7 IgG and IgA in plasma and fecal supernatants

Free soluble IgA and IgG in plasma and fecal supernatants were analyzed by ELISA to detect anti‐STEC O157:H7 antibodies, using intact formalin‐killed STEC O157:H7 as the coating antigen, as previously described^14^, ^50^. Briefly, 96‐well MaxiSorp plates (Greiner Bio‐One GmbH) coated with STEC O157:H7 overnight were washed with PBS containing 0.05% Tween20 (PBS‐T) and blocked with 3% milk powder (Sigma) in PBS (blocking buffer) for 1.5 h at room temperature. After washing with PBS‐T, plasma or fecal supernatant samples were added in serial dilutions and incubated overnight at 4°C. Then, wells were washed with PBS‐T and treated with HRP‐conjugated goat anti‐mouse IgA (1/3000; Invitrogen) or HRP‐conjugated goat anti‐mouse IgG (1/3000; Invitrogen) diluted in blocking buffer. The plates were incubated for 1.5 h at room temperature. After washing, 2 mg/ml o‐phenylenediamine (Sigma‐Aldrich) and 0.3% H₂O₂ were added in 0.1 M citrate‐0.2 M HPO₄^−^² buffer (pH 5.0). The reaction was stopped by adding 1 M H₂SO₄, and absorbance at OD_492_ was measured using an Asys UVM340 microplate reader (Biochrom Ltd.). Antibody levels were expressed as OD_492_ units, calculated as the value obtained for each sample minus the OD_492_ for nonspecific binding at the same dilution. When values were expressed as titers, they correspond to the inverse of the last sample dilution that yields an OD_492_ significantly higher than that of control samples. Data of IgG‐anti STEC O157:H7 in plasma, when required, were transformed into the base‐2 logarithm (log₂), to comply with normality assumptions and apply a parametric statistical test.

#### Determination of the anti‐B subunit of Stx2 (Stx2B) IgG in plasma

Stx2B‐specific IgG in plasma was analyzed by ELISA, following previously described protocols^23^, ^29^. In brief, 96‐well MaxiSorp plates (Greiner Bio‐One) were coated overnight at 4°C with 5 µg/ml of purified Stx2B in 15 mM carbonate‐25 mM bicarbonate buffer (pH 9.6). The wells were washed with PBS‐T and blocked with blocking buffer for 1.5 h at room temperature. After washing with PBS‐T, plasma samples were added in serial dilutions and incubated overnight at 4°C. After washing with PBS‐T, wells were incubated with HRP‐conjugated goat anti‐mouse IgG (1/3000, Invitrogen). The chromogenic substrate was added, and the reaction was carried out as described above. Antibody levels were expressed as OD_492_, calculated as the sample value minus the OD_492_ for nonspecific binding.

#### Determination of anti‐H7 IgG and IgA in plasma and fecal supernatants

Anti‐H7‐specific IgG and IgA in plasma and fecal supernatants were quantified by ELISA coating 96‐well plates with 5 μg/ml of purified H7 in PBS (pH 7.4) as previously described^29^. After blocking, serial dilutions of plasma or fecal supernatants were added and incubated overnight at 4°C. Then, plates were washed and incubated with HRP‐conjugated goat anti‐mouse IgG (1/3000; Invitrogen) or HRP‐conjugated goat anti‐mouse IgA (1/3000; Invitrogen). The reaction was conducted as described in above sections. Results were expressed as anti‐H7 titers, which were calculated as the last sample dilution that presents an OD_492_ higher and statistically significantly different from the control samples.

### Neutralizing activity of anti‐STEC O157:H7 Abs in plasma from infected BALB/c mice

#### Bacterial motility

1 × 10^3^ CFU of STEC O157:H7 were incubated with plasma from non‐infected or infected BALB/c mice (diluted 1/10 in 0.1 ml PBS) for 18 h at 4°C. An additional control of bacteria incubated without plasma was included. After incubation, bacteria were washed by centrifugation (13,300 rpm for 5 min) and finally resuspended in 0.1 ml of PBS. Four microliter of each bacterial suspension was seeded in triplicate at the center of soft agar plates (1% peptone casein acid, 0.25% agar, and 0.25% NaCl), and after 24 and 48 h of incubation at 37°C, the diffusion halos were measured to determine the bacterial motility in millimeter (mm).

#### Bacterial adhesion to intestinal epithelial cells

3 × 10^7^ CFU of STEC O157:H7 were incubated with plasma from non‐infected or infected BALB/c mice (diluted 1/10 in 0.1 ml PBS) for 18 h. The additional control without plasma was included in these studies. After incubation, bacteria were washed by centrifugation, and bacterial pellets were resuspended in 0.3 ml of minimal essential medium (MEM)‐HEPES (Gibco) supplemented with 0.1% (w/v) glucose and 250 nM Fe(NO_3_)_3_ (complete medium) and placed in triplicate (0.1 ml/well) into 12‐well culture plates to induce infection of a monolayer of 250,000 Caco‐2 cells/well (in a final volume of 0.5 ml complete medium) as previously described^46^. Briefly, plates were centrifuged at 1000*g* for 5 min at 10°C to synchronize bacterial adhesion and cultured at 37°C with 5% CO_2_ for 3 h. After supernatants containing non‐adherent bacteria were discarded, 0.2 ml of PBS containing 0.1% Triton X‐100 was added to each well and the cells were scraped off. Suspensions were serially diluted in PBS and plated onto LB agar plates, incubated overnight at 37°C, and colonies were counted. The percentage of adherence was calculated as (number of viable STEC O157:H7 recovered after lysis of eukaryotic cells/number of initial viable STEC O157:H7 placed to infect eukaryotic cells) × 100%.

#### Bacterial growth

1 × 10^3^ CFU of STEC O157:H7 were incubated with plasma (diluted 1/10 in 0.1 ml PBS) from non‐infected or infected BALB/c mice for 18 h. The additional control without plasma was included in these studies. After incubation, bacteria were washed by centrifugation and placed into 6‐well culture plates containing 3 ml of TSB. Bacterial growth was performed in a multimode plate reader (Varioskan Lux, Thermo Fisher, USA) for 18 h at 37°C, measuring OD_600_ every 20 min. At time zero, the quantification of the number of viable bacteria (CFU/ml) of each suspension was performed by plating dilutions onto LB agar plates to rule out differences in the number of initial live bacteria per treatment.

### Statistical methods

Data were expressed as the mean ± SEM if they met the assumptions of normality (Gaussian distribution, verified using the Shapiro–Wilk test) and homogeneity of variance (assessed by Levene's test or *F*‐test, as appropriate). When both assumptions were satisfied, statistical analysis was performed using Student's *t*‐test, one‐way ANOVA, or two‐way ANOVA, followed by Tukey's posttest, depending on the number of experimental groups. If the assumptions were not met, data were expressed as the median ± interquartile range and analyzed using non‐parametric tests, such as the Mann–Whitney *U* test or Kruskal–Wallis test, based on the number of groups. Survival curves were constructed for significance using the Mantel–Cox test. All analyses were conducted using Prism 8.0 software (GraphPad) or InfoStat software (Córdoba, Argentina), with graphical representations created in Prism 8.0. Statistical significance was defined as *p* < 0.05.

## AUTHOR CONTRIBUTIONS

**Alan Mauro Bernal**: Conceptualization; investigation; writing—original draft. **Fernando Nicolás Sosa**: Data curation; methodology; resources. **Yina María Carpintero‐Polanco**: Data curation; methodology; resources. **Camila Dara Cancino**: Data curation; methodology; resources. **Romina Jimena Fernández‐Brando**: Methodology; supervision. **María Victoria Ramos**: Methodology; supervision. **Ariel Podhozer**: Data curation; methodology; resources. **Agustina Errea**: Data curation; methodology; resources. **Martín Rumbo**: Methodology; supervision. **Marina Sandra Palermo**: Conceptualization; funding acquisition; supervision; writing—review and editing.

## ETHICS STATEMENT

The Institutional Animal Care and Use Committee at IMEX‐CONICET‐Academia Nacional de Medicina approved all procedures in accordance with the principles set forth in the Guide for the Care and Use of Laboratory Animals (protocol number 86/2021)^51^. The health and behavior of mice were assessed twice a day. Any unnecessary pain, discomfort, or injury to animals was avoided. Mice that became moribund (with a weight loss greater than 20% of its initial value and/or plasma urea levels higher than 150 mg%) were humanely euthanized by intraperitoneal administration of ketamine (75 mg/kg mouse) and xylazine (15 mg/kg mouse) by subsequent cervical dislocation. Institutional Animal Care & Use Committee (IACUC) guidelines were used to define humane endpoints.

## CONFLICT OF INTERESTS

The authors declare no conflicts of interests.

## Supporting information

Supplementary data.

## Data Availability

The raw data supporting the conclusions of this article will be made available by the authors, without undue reservation.
