# Supplementary material for: Neutralizing antibodies in the intestinal mucosa are essential to control gastrointestinal infection by Shiga toxin‐producing Escherichia coli
Source: mLife. 2025 Aug 25;4(4):409–22. doi: 10.1002/mlf2.70026 (PMC12396203; doi:10.1002/mlf2.70026)
Supplement: Supplementary file 1 — Supplementary data. [file MLF2-4-409-s001.docx]

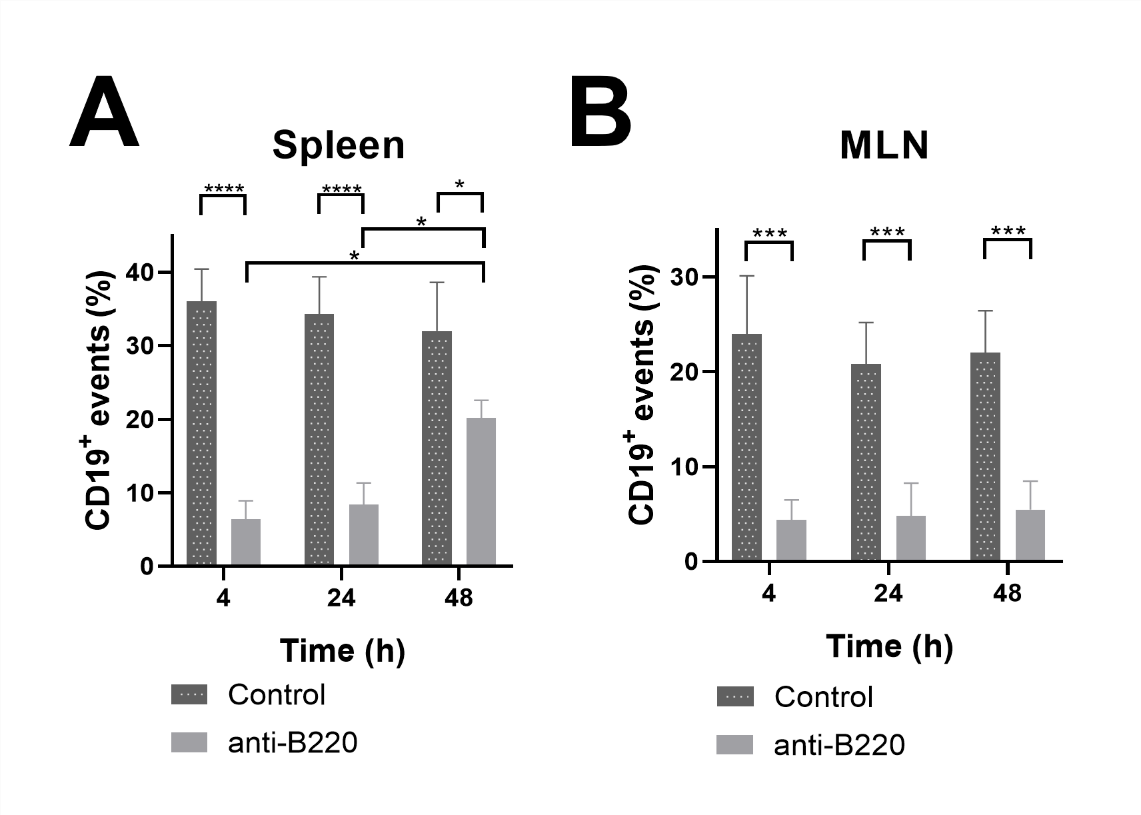


**Figure S1. Depletion of B lymphocytes in spleen and MLN.** BALB/c mice at weaning were treated with PBS or anti-B220 monoclonal Ab (2.8 mg/mouse i.v.) and euthanized after 4, 24 and 48 h to determine the percentage of B lymphocytes in spleen and MLN by flow cytometry, as described in Materials and Methods, identifying this population by using FITC-coupled anti-mouse CD19. **(A) Percentage of B lymphocytes in spleen. (B) Percentage of B lymphocytes in MLN.** Each bar shows the mean ± SEM of 3 mice for each treatment. Data were analyzed by two-way ANOVA with Tukey’s post-test. *p<0.05, ***p<0.001, ****p<0.0001.


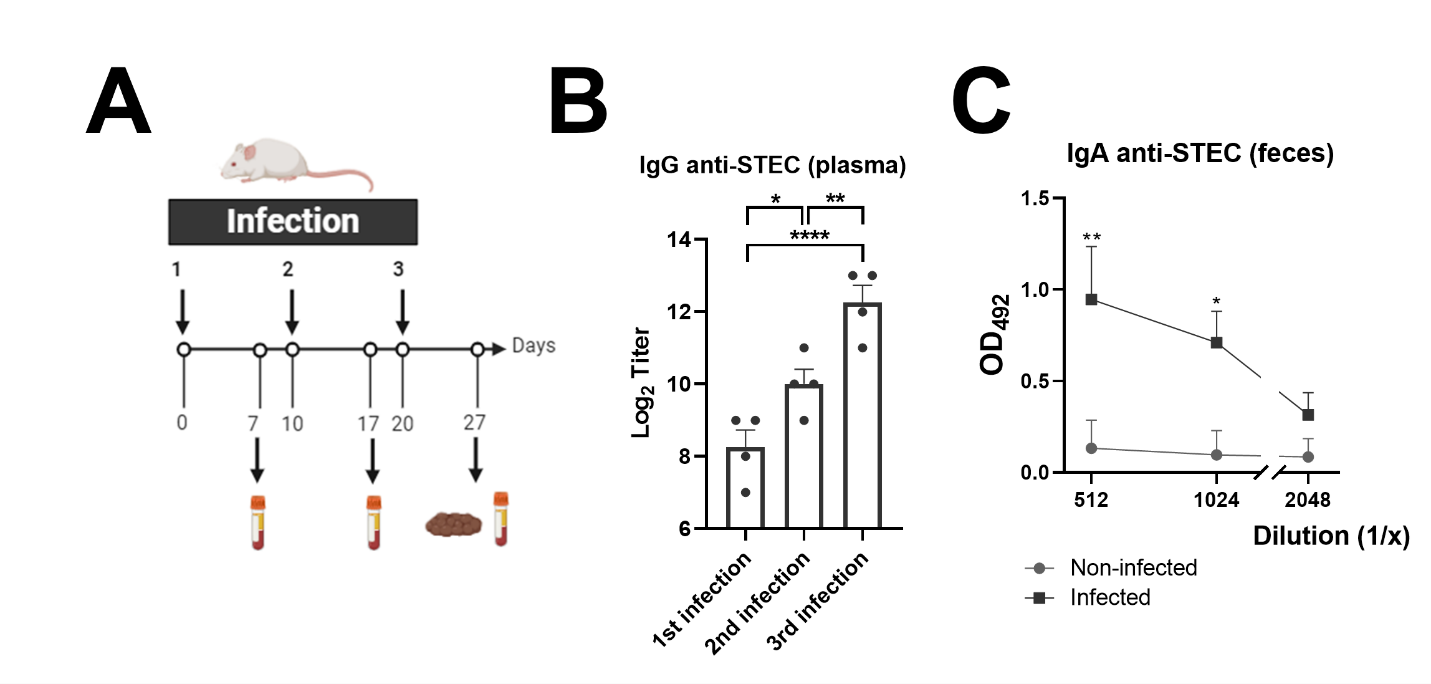


**Figure S2. Reinfection of BALB/c mice.** BALB/c mice were g.i. infected with a non-lethal dose of STEC O157:H7 (1x10^10^ CFU/mouse) and re-challenged twice (5x10^10^ CFU/mouse) at 10-day intervals. Mice were bled 7 days after each challenge to determine plasma anti-STEC O157:H7 Ab titer by ELISA, as described in Materials and Methods. Seven days after the last challenge, mice were euthanized, and plasma and feces collected to determine anti-STEC O157:H7 Ab titers, obtained as the last dilution of the sample (plasma or feces) showing a significantly higher OD492 in comparison with the control sample at the same dilution. **(A) Reinfection scheme. (B) Anti-STEC O157:H7 IgG titer in plasma expressed as the base 2 logarithm (Log2).** Data were log transformed to base 2 to comply with normality assumptions and apply a one-way ANOVA with Tukey’s post-test. *p<0.05, **p<0.01, ****p<0.0001. **(C) Anti-STEC O157:H7 IgA in feces represented as OD492 vs the inverse of serial sample dilution.** Data were analyzed by two-way ANOVA with Tukey’s post-test *p<0.05, **p<0.01. Each bar or point represents the mean ± SEM of 4 mice/treatment.

**
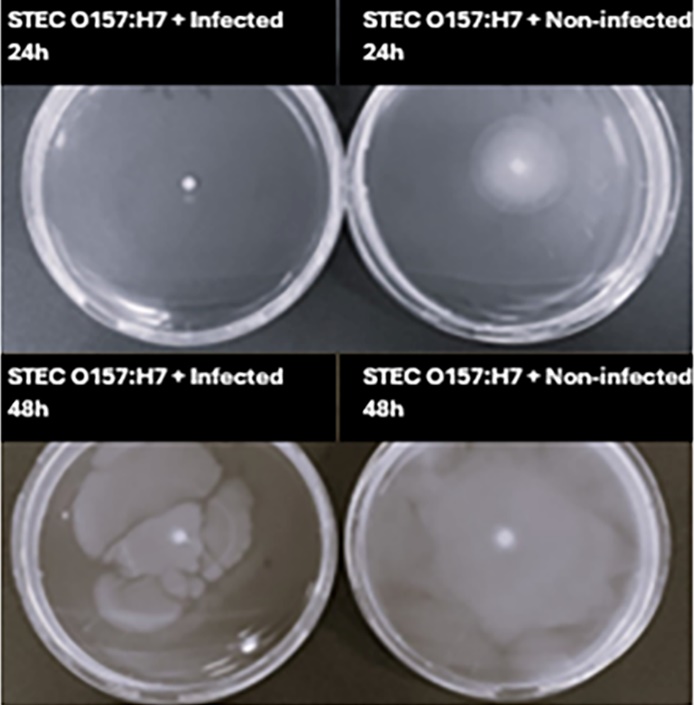
**

**Figure S3.** STEC O157:H7 were incubated with plasma (1/10) from infected or non-infected mice for 18 h and seeded by triplicate in the center of soft agar plates as described in Materials and Methods to assay bacterial motility. **Representative images** of soft agar plates **showing diffusion halos** at 24 and 48 h of STEC O157:H7.
